# A novel missense variant in *MYO3A* is associated with autosomal dominant high‐frequency hearing loss in a German family

**DOI:** 10.1002/mgg3.1343

**Published:** 2020-06-10

**Authors:** Julia Doll, Michaela A. H. Hofrichter, Paulina Bahena, Alfred Heihoff, Dennis Segebarth, Tobias Müller, Marcus Dittrich, Thomas Haaf, Barbara Vona

**Affiliations:** ^1^ Institute of Human Genetics Julius Maximilians University Würzburg Germany; ^2^ Joint Practice of Pediatrics Regensburg Germany; ^3^ Institute of Clinical Neurobiology University Hospital Würzburg Würzburg Germany; ^4^ Institute of Bioinformatics Julius Maximilians University Würzburg Germany; ^5^ Tübingen Hearing Research Centre Department of Otolaryngology ‐ Head and Neck Surgery Eberhard Karls University Tübingen Germany

**Keywords:** autosomal dominant nonsyndromic hearing loss, *MYO3A*, myosin IIIA, progressive hearing loss, sensorineural hearing loss

## Abstract

**Background:**

*MYO3A*, encoding the myosin IIIA protein, is associated with autosomal recessive and autosomal dominant nonsyndromic hearing loss. To date, only two missense variants located in the motor‐head domain of *MYO3A* have been described in autosomal dominant families with progressive, mild‐to‐profound sensorineural hearing loss. These variants alter the ATPase activity of myosin IIIA.

**Methods:**

Exome sequencing of a proband from a three‐generation German family with prelingual, moderate‐to‐profound, high‐frequency hearing loss was performed. Segregation analysis confirmed a dominant inheritance pattern. Regression analysis of mean hearing level thresholds per individual and ear was performed at high‐, mid‐, and low‐frequencies.

**Results:**

A novel heterozygous missense variant c.716T>C, p.(Leu239Pro) in the kinase domain of *MYO3A* was identified that is predicted in silico as disease causing. High‐frequency, progressive hearing loss was identified.

**Conclusion:**

Correlation analysis of pure‐tone hearing thresholds revealed progressive hearing loss, especially in the high‐frequencies. In the present study, we report the first dominant likely pathogenic variant in *MYO3A* in a European family and further support *MYO3A* as an autosomal dominant hearing loss gene.

## Graphical abstract

A novel heterozygous missense variant c.716T>C, p.(Leu239Pro) in the kinase domain of *MYO3A* was identified in a German family with high‐frequency autosomal dominant sensorineural hearing loss. We provide in‐depth characterization of the hearing loss progression and confirmatory evidence of *MYO3A* being associated with autosomal dominant non‐syndromic hearing loss.

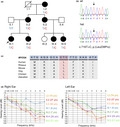

## 1 INTRODUCTION

Hearing loss (HL) belongs to the most common sensory disorders in humans and shows a prevalence of 1–3 in 1,000 newborns (Vona, Nanda, Hofrichter, Shehata‐Dieler, & Haaf, 2015). Nonsyndromic autosomal dominant hearing loss (DFNA) was first described in 1997 in a Caucasian family with profound deafness caused by a mutation in *GJB2* (Kelsell et al., 1997). Since then, approximately 50 autosomal dominant HL genes have been identified, with 13 genes showing both dominant and recessive inheritance patterns (http://hereditaryhearingloss.org). *MYO3A* (OMIM #606808), a 33‐exon gene on chromosome 10p12.1, was first associated with autosomal recessive HL (DFNB30) in 2002. Affected individuals from a Jewish family showed bilateral, progressive, high‐frequency HL beginning in the second decade of life caused by three different recessive loss‐of‐function variants in *MYO3A* (Walsh et al., 2002). Since then, several additional recessive loss‐of‐function and missense variants with a wide range of HL characteristics were identified in patients with nonsyndromic HL (Choi et al., 2013; Miyagawa, Naito, Nishio, Kamatani, & Usami, 2013; Qu et al., 2016; Sommen et al., 2016; Wu et al., 2015). In 2016, the inheritance pattern of HL due to variants in *MYO3A* expanded with the discovery of a missense variant in an African‐American family with progressive, postlingual childhood onset HL (Grati et al., 2016). Later, in 2018, two Brazilian families with late‐onset nonsyndromic HL were identified with the same segregating missense variant (Dantas et al., 2018). To date, only three autosomal dominant families with mild‐to‐profound, progressive HL have been identified with two different segregating missense variants in *MYO3A* (Dantas et al., 2018; Grati et al., 2016).

In vertebrates, two different isoforms have been identified; the longer Myo3A isoform (209 kDa) and a shorter Myo3B isoform (155 kDa), that are both expressed in the retina and testis (Dose et al., 2003). The encoded myosin IIIA protein consists of a N‐terminal kinase domain, a highly conserved motor‐head domain, followed by three calmodulin binding (IQ) motifs and a C‐terminal actin‐binding domain (3THD‐II) (Dose & Burnside, 2000; Salles et al., 2009). Both previously described dominant variants in *MYO3A* are located in the motor‐head domain and are thought to affect the ATPase activity of the gene (Dantas et al., 2018; Grati et al., 2016). Expression is present in the retina (Dose & Burnside, 2000) and the inner ear of mammals (Schneider et al., 2006; Walsh et al., 2002), specifically at the tips of both inner and outer hair cells in all stereocilia rows, as well as in vestibular hair cell stereocilia in mice (Walsh et al., 2011). Mutant mice homozygous for a nonsense allele, show progressive, high‐frequency HL, advancing to all frequencies over time (Walsh et al., 2011).

We describe the first European family with dominant, moderate‐to‐profound, high‐frequency sensorineural HL with a novel heterozygous missense variant c.716T>C, p.(Leu239Pro) in *MYO3A* (NM_017433.4). This finding supports and consolidates the association of autosomal dominant HL due to variants in this gene. Due to a relative lack of studies on the genetic basis of autosomal dominant HL and the challenging occurrence of a typical later age of onset, characterization of large families is important for unraveling the distinction between autosomal recessive and dominant alleles in the personalized medicine era.

## 2 METHODS

### 2.1 Ethical compliance

Written informed consent was obtained from the family and all procedures were approved by the Ethics Commission of the University of Würzburg (46/15, approval date: 31 March 2015).

### 2.2 Clinical evaluation

We recruited the genomic DNA from a three‐generation German family with seven affected (I.2, II.2, II.3, III.1, III.2, III.4, III.5) and one unaffected individual (II.1). Audiological testing, including pure‐tone audiometry, was done for all affected family members and the unaffected family member III.3 complying with guidelines described by Mazzoli et al. (2003).

### 2.3 Genomic analysis and exome sequencing

Genomic DNA from participating affected and unaffected individuals was extracted from whole blood. We excluded pathogenic variants in the most common gene, *GJB2*, by diagnostic Sanger sequencing of the index patient. Exome sequencing of the index patient (III.1) was performed. Exome library preparation was performed with the Nextera Rapid Capture Exome kit (Illumina) according to manufacturer's instructions and paired‐end sequenced (2 × 76 bp) with a v2 high‐output reagent kit with the NextSeq500 sequencer (Illumina). The human reference genome GRCh37 (hg19) was used for data alignment.

### 2.4 Exome analysis

Single nucleotide variants (SNVs) and small indels (<15bp) were analyzed using GensearchNGS software (PhenoSystems SA) and our in‐house bioinformatics pipeline. Variant filtering followed an alternate allele frequency present at >20% and a minor allele frequency <0.01. Reads were aligned to hg19 using BWA (Li & Durbin, 2010) and the GATK toolkit according to GATK best practice (DePristo et al., 2011). Variants were filtered by quality based on the VQSLOD score that indicates the log odds ratio of the probability that each variant is true (McKenna et al., 2010). Population‐specific allele frequencies were assessed using gnomAD (Karczewski et al., 2019). PolyPhen‐2 (Adzhubei et al., 2010), MutationTaster (Ng & Henikoff, 2001) and SIFT (Schwarz, Cooper, Schuelke, & Seelow, 2014) were used to analyze the effects of SNVs, as well as the Deafness Variation Database (DVD) (Azaiez et al., 2018) and the Human Gene Mutation Database (HGMD) (Stenson et al., 2003) for variant interpretation. Potential splicing effects of variants were classified by in silico prediction tools such as SpliceSiteFinder‐like (Shapiro & Senapathy, 1987), MaxEntScan (Yeo & Burge, 2004), NNSPLICE (Reese, Eeckman, Kulp, & Haussler, 1997), Genesplicer (Pertea, Lin, & Salzberg, 2001), and Human Splicing Finder (Desmet et al., 2009). CNVs were investigated using the eXome Hidden Markov Model (XHMM, version 1.0) approach (Fromer & Purcell, 2014).

### 2.5 Sanger validation and segregation of the *MYO3A* c.716T>C variant

PCR amplification and Sanger sequencing of the genomic DNA of the index patient, as well as affected and unaffected family members was performed to validate the c.716T>C missense variant in *MYO3A* (NM_017433.4). Primers were designed with Primer3 (Untergasser et al., 2012) (F: 5′‐TACTAGGTGATTGCATGTGAACAG‐3′, and R: 5′‐TGAAGAGCATGATGAACACTTGG‐3′) and standard cycling conditions were used. An ABI 3130xl 16‐capillary sequencer (Life Technologies) was used for amplicon sequencing and the data were analyzed with the Gensearch 4.3 software (PhenoSystems SA).

### 2.6 Protein modeling prediction of wild type MYO3A

The secondary protein structure at amino acid position 239 (Leu) of wild type MYO3A was predicted in silico with I‐TASSER (Yang et al., 2015). C‐scores ranging from −5 to 2 indicate the confidence of the predicted models, where a C‐score of a higher value indicates a model with a higher confidence.

### 2.7 Correlation analysis

We calculated the mean hearing level threshold per patient and ear at high‐ (4–8 kHz), mid‐ (1–3 kHz), and low‐ (0.125–0.5 kHz) frequencies. Pearson's correlation between age at audiometric examination and mean hearing level threshold was calculated using OriginPro 2019b (OriginLab Corporation) (Figure S1).

## 3 RESULTS

### 3.1 Clinical presentation of the German family

**FIGURE 1:**
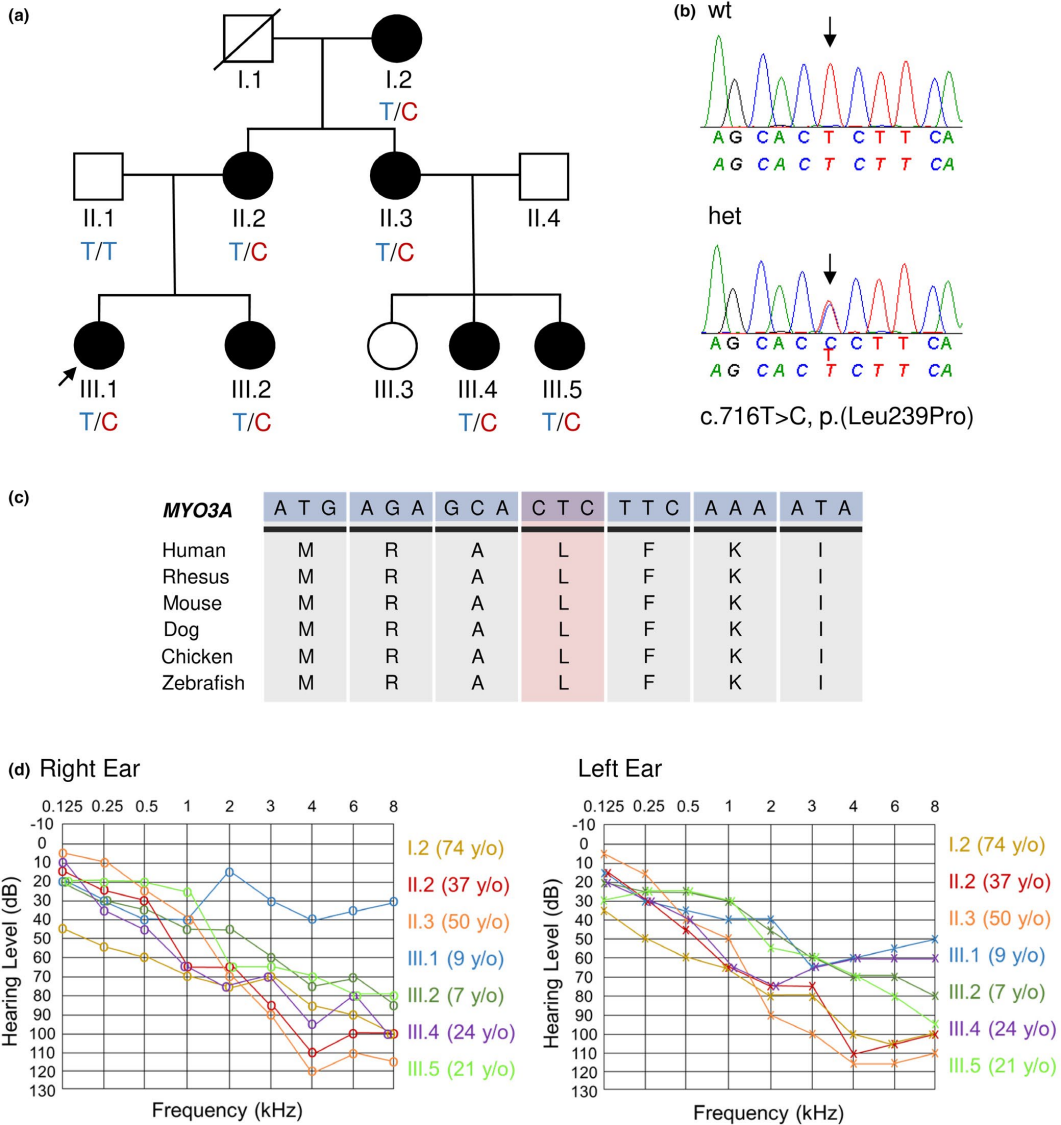
Pedigree of the German family, segregation, and conservation of the novel missense variant, and audiograms of affected family members. (a) A three‐generation German family with seven affected individuals are marked with black symbols and the index patient marked with an arrow. The two unaffected family members are marked with white symbols. Heterozygous, affected individuals with the c.716T>C, p.(Leu239Pro) variant are marked with “T/C”. The wild type, unaffected individual is marked with “T/T”. (b) Chromatograms showing the wild type Sanger sequence (wt, top) and the heterozygous sequence (het, below) of the c.716T>C variant. (c) Conservation of the amino acid position 239 (L) and flanking regions across different species. (d) Right and left ear pure‐tone audiogram thresholds (air conduction) of affected family members (I.2, II.2, II.3, III.1, III.2, III.4, III.5) and the age at the time of examination

We present a three‐generation German family with prelingual, bilateral, sensorineural HL (Figure 1a). All affected individuals (I.2, II.2, II.3, III.1, III.2, III.4, III.5) showed high‐frequency HL ranging from moderate to profound in severity (Figure 1d). Individual III.3 underwent audiometry and revealed normal hearing (data not shown). Our data show a clear correlation between the average thresholds at high‐ (4–8 kHz) and mid‐ (1–3 kHz) frequencies and the age at audiometric examination for both ears, indicating a progressive HL (Pearson's r: 0.62 (right ear), 0.76 (left ear) for high‐frequencies; 0.71 (right ear), 0.83 (left ear) for mid‐frequencies) (Figure S1). All affected individuals use hearing aids and show a good hearing outcome. After clinical examination, additional symptoms and risk factors for hearing loss such as infections and trauma were excluded. Tinnitus was not reported for all affected individuals.

### 3.2 Identification and analysis of a novel missense variant in *MYO3A*

The index patient (III.1) underwent exome sequencing and bioinformatics analysis that included 174 deafness‐associated genes (Table S1) as an initial approach to screen variants in clinically relevant hearing loss‐associated genes that was followed by an exome‐wide analysis. A novel heterozygous missense variant c.716T>C, p.(Leu239Pro) in exon 8 of the gene *MYO3A* (NCBI Reference Sequence: NM_017433.4) was identified that was predicted in silico as disease causing and resulted in a putative pathogenic amino acid exchange according to several in silico tools (PolyPhen‐2, MutationTaster, and SIFT). The variant is classified as “likely pathogenic” according to the ClinGen hearing loss working group expert specification (Oza et al., 2018). The variant affects a highly conserved nucleotide and amino acid (Figure 1c) that is part of the catalytic kinase domain of the encoded MYO3A protein. Protein modeling prediction of wild type MYO3A indicated an alpha‐helix at amino acid position 239 (Leu), based on the two predicted models with the highest C‐score (−1.82, −2.00). Segregation testing of the c.716T>C variant followed a dominant inheritance pattern (Figure 1a,b). Bioinformatics analysis in 174 Hl genes excluded additional potentially disease‐causing variants and copy number variations (CNVs) that could resolve the phenotype of the family. The c.716T>C variant has been submitted to the Leiden Open Variation Database version 3 (LOVD v.3.0) under variant ID 0000660455.

## 4 DISCUSSION

**FIGURE 2:**
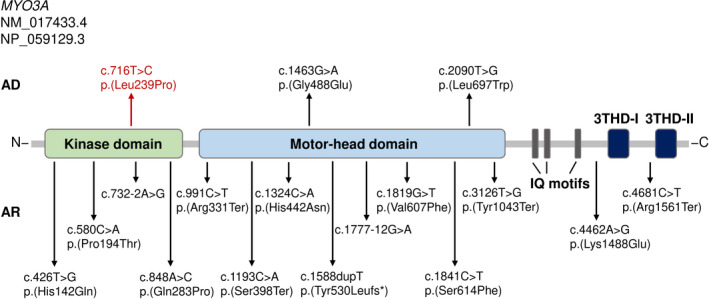
Summary of all identified recessive and dominant HL variants in *MYO3A* (NM_017433.4, NP_059129.3). The encoded myosin IIIA protein is composed of a N‐terminal catalytic kinase domain, a motor‐head domain, three calmodulin binding (IQ) motifs, a C‐terminal domain containing a N‐terminal unit (3THD‐I) and an actin‐binding domain (3THD‐II). Previously described autosomal dominant (AD) missense variants are shown above in black and the newly identified variant c.716T>C, p.(Leu239Pro) is marked in red. Already identified autosomal recessive (AR) variants are shown below

*MYO3A* belongs to the unconventional myosins (class III) of the large myosin superfamily (Dose & Burnside, 2000). They mediate crucial cellular functions such as signal transduction, cell movement, and vesicle trafficking (Mermall, Post, & Mooseker, 1998). Grati et al. described the first autosomal dominant mutation (p.(Gly488Glu), Figure 2) resulting in an amino acid substitution in the motor‐head domain that modifies the ATPase activity of MYO3A at the hair cell stereocilia tips. These authors also showed that MYO3A interacts with PCDH15 (protocadherin 15), a crucial component of the mechanoelectrical transduction (MET) complex (Grati et al., 2016). The two previously described dominant variants in an African‐American (c.1463G>A, p.(Gly488Glu)) and two Brazilian families (c.2090T>G, p.(Leu697Trp)) are both located in the motor‐head domain of the myosin IIIa protein (Figure 2). The affected individuals showed nonsyndromic, bilateral, progressive HL, ranging from mild to profound in severity. The age of onset varied between early childhood (postlingual) and an average age of onset of 30 to 32 years (Table S2) (Dantas et al., 2018; Grati et al., 2016). Furthermore, one congenital case was reported in one of the Brazilian families (Dantas et al., 2018). Interestingly, there is a great variability regarding the age of onset and HL severity in families with previously described recessive variants in *MYO3A*, ranging from congenital to late‐onset HL and a moderate‐to‐profound degree of severity. Additionally, there is no apparent clustering of recessive and dominant variants in a certain protein domain, such as the kinase or motor‐head domain (Table S2).

The affected individuals in the German family we describe, all showed nonsyndromic, prelingual, progressive HL (Figure 1b,c), especially impacting high‐ (4–8 kHz) and mid‐ (1–3 kHz) frequencies (Figure S1). Progressive HL is observed in other autosomal dominant *MYO3A* families. As previously described, *MYO3A* variants that are implicated in an autosomal dominant inheritance confer a dominant‐negative effect, reducing actin protrusion initiation, and elongation activity of the encoded protein in inner ear hair cell stereocilia (Dantas et al., 2018). Dominant‐negative effects are characterized by the adverse assembly of wild type and mutant protein subunits, preventing correct protein functionality and are frequently involved in various human diseases (Bergendahl et al., 2019; Herskowitz, 1987; Marziano, Casalotti, Portelli, Becker, & Forge, 2003). The c.716T>C, p.(Leu239Pro) missense variant in the German family is the first dominant variant that is located in the kinase domain of the gene (Figure 2). In vitro analysis suggests that a functional kinase domain of the MYO3A protein is important for proper regulation of actin dynamics and stability of actin bundles at filopodial tips (Quintero et al., 2010). The heterozygous variant results in an amino acid exchange from leucine to proline that is predicted to be disease causing in silico. The exchange from a branch‐chain leucine to a cyclic proline possibly mediates the disruption of the present alpha‐helix at amino acid position 239 (Roy, Kucukural, & Zhang, 2010; Yang et al., 2015; Zhang, 2008) and results in a structural protein change (Bajaj et al., 2007; Cordes, Bright, & Sansom, 2002). Although it was not directly tested, it is suspected that the potentially defective MYO3A protein also interacts with the existing wild type protein via a dominant‐negative mechanism and is responsible for the HL phenotype in the German family.

Compared to its recessive counterpart, many dominant forms of HL lack in‐depth clinical characterization. Clinical data from large families are essential to discriminate progression, which is of high interest to directing current and future treatment modalities. Several genes, including *MYO3A*, lack substantial clinical validity through lack of replication evidence. Here, we describe the first European family with a novel dominant variant in *MYO3A*, thus, providing further evidence for the association of this gene with an autosomal dominant HL phenotype.

## CONFLICT OF INTEREST

The authors declare no conflict of interest.

## AUTHOR CONTRIBUTIONS

Conceptualization, T.H., B.V.; Manuscript drafting, J.D., T.H., B.V.; Ascertained family and obtained clinical data, M.A.H.H., P.B., A.H., J.D., B.V.; Supervision, T.H., B.V.; Exome sequencing and segregation analysis, J.D., M.A.H.H., B.V.; Bioinformatics support, T.M., M.D.; Protein modeling, J.D., B.V.; Correlation analysis, D.S., J.D.; All authors participated in final review and editing of the manuscript. All authors have read and agreed to the published version of the manuscript.

## Supporting information

Fig S1

Table S1

Table S2

## Data Availability

The data that support the findings of this study are available on request from the corresponding author. The data are not publicly available due to privacy or ethical restrictions. The variant was deposited in LOVD3 under variant ID 0000660455.
