# Supplementary figures and images for: A novel missense variant in MYO3A is associated with autosomal dominant high‐frequency hearing loss in a German family
Source: Mol Genet Genomic Med. 2020 Jun 10;8(8):e1343. doi: 10.1002/mgg3.1343 (PMC7434730; doi:10.1002/mgg3.1343)

A

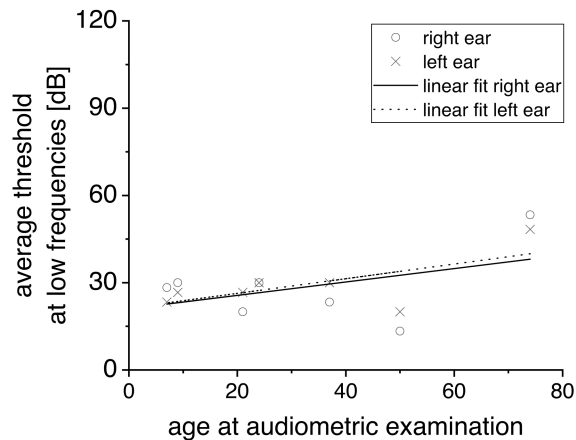

B

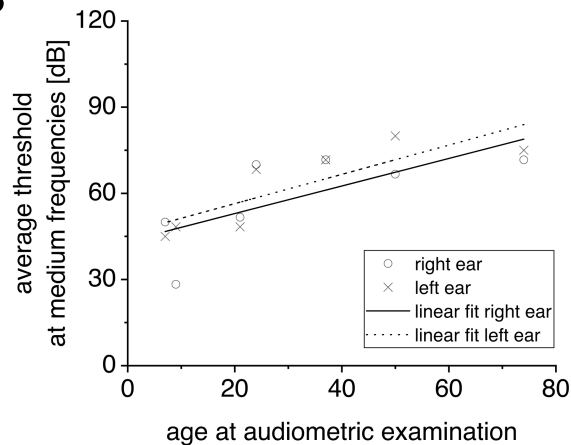

C

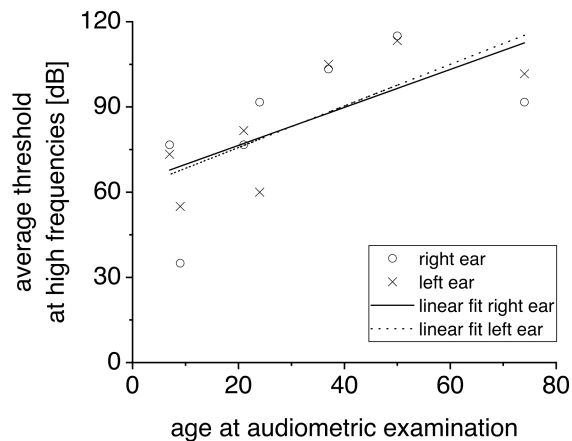

Supplement: Supplementary file 1 — Fig S1 [file MGG3-8-e1343-s001.pdf]
