# Supplementary material for: A novel missense variant in MYO3A is associated with autosomal dominant high‐frequency hearing loss in a German family
Source: Mol Genet Genomic Med. 2020 Jun 10;8(8):e1343. doi: 10.1002/mgg3.1343 (PMC7434730; doi:10.1002/mgg3.1343)
Supplement: Supplementary file 2 — Table S1 [file MGG3-8-e1343-s002.docx]

**Table S1:** List of hearing loss-associated genes and their corresponding OMIM numbers.

| **Gene** | **OMIM #** | **Gene** | **OMIM #** |
| --- | --- | --- | --- |
| *ABHD12* | 613599 | *COCH* | 603196 |
| *ACTG1* | 102560 | *COL11A1* | 120280 |
| *ADCY1* | 103072 | *COL11A2* | 120290 |
| *ADGRV1* | 602851 | *COL2A1* | 120140 |
| *AIFM1* | 300169 | *COL4A3* | 120070 |
| *ARSG* | 610008 | *COL4A4* | 120131 |
| *ATP1A3* | 182350 | *COL4A5* | 303630 |
| *ATP2B2* | 108733 | *COL4A6* | 303631 |
| *BDP1* | 607012 | *COL9A1* | 120210 |
| *BSND* | 606412 | *COL9A2* | 120260 |
| *BTD* | 609019 | *COL9A3* | 120270 |
| *CABP2* | 607314 | *CRYM* | 123740 |
| *CCDC50* | 611051 | *DCDC2* | 605755 |
| *CD164* | 603356 | *DIABLO* | 605219 |
| *CDC14A* | 603504 | *DIAPH1* | 602121 |
| *CDH23* | 605516 | *DIAPH3* | 614567 |
| *CEACAM16* | 614591 | *DMXL2* | 612186 |
| *CEP250* | 609689 | *EDN3* | 131242 |
| *CEP78* | 617110 | *EDNRB* | 131244 |
| *CHD7* | 608892 | *ELMOD3* | 615427 |
| *CHST3* | 603799 | *EPS8* | 600206 |
| *CIB2* | 605564 | *EPS8L2* | 614988 |
| *CISD2* | 611507 | *ERAL1* | 607435 |
| *CLDN14* | 605608 | *ESPN* | 606351 |
| *CLIC5* | 607293 | *ESRP1* | 612959 |
| *CLPP* | 601119 | *ESRRB* | 602167 |
| *CLRN1* | 606397 | *EYA1* | 601653 |

| **Gene** | **OMIM #** | **Gene** | **OMIM #** |
| --- | --- | --- | --- |
| *EYA4* | 603550 | *KCNQ4* | 603537 |
| *FAM65B* | 611410 | *KIAA0391* | 609947 |
| *FGF3* | 164950 | *KIT* | 164920 |
| *FGFR1* | 136350 | *KITLG* | 184745 |
| *FGFR2* | 176943 | *KMT2D* | 602113 |
| *FITM2* | 612029 | *LARS2* | 604544 |
| *FOXI1* | 601093 | *LHFPL5* | 609427 |
| *GAB1* | 604439 | *LMX1A* | 600298 |
| *GATA3* | 131320 | *LOXHD1* | 613072 |
| *GIPC3* | 608792 | *LOXL3* | 607163 |
| *GJB2* | 121011 | *LRP5* | 603506 |
| *GJB3* | 603324 | *LRTOMT* | 612414 |
| *GJB6* | 604418 | *MARVELD2* | 610572 |
| *GPSM2* | 609245 | *MASP1* | 600521 |
| *GRHL2* | 608576 | *MCM2* | 116945 |
| *GRXCR1* | 613283 | *MET* | 164860 |
| *GRXCR2* | 615762 | *METTL13* | 617987 |
| *GSDME* | 608798 | *MIR96* | 611606 |
| *HARS* | 142810 | *MITF* | 156845 |
| *HARS2* | 600783 | *MPZL2* | 604873 |
| *HGF* | 142409 | *MSRB3* | 613719 |
| *HOMER2* | 604799 | *MYH14* | 608568 |
| *HSD17B4* | 601860 | *MYH9* | 160775 |
| *IFNLR1* | 607404 | *MYO15A* | 602666 |
| *ILDR1* | 609739 | *MYO3A* | 606808 |
| *KARS* | 601421 | *MYO6* | 600970 |
| *KCNE1* | 176261 | *MYO7A* | 276903 |
| *KCNJ10* | 602208 | *NARS2* | 612803 |
| *KCNQ1* | 607542 | *NISCH* | 615507 |
| **Gene** | **OMIM #** | **Gene** | **OMIM #** |
| *OSBPL2* | 606731 | *SLC44A4* | 606107 |
| *OTOA* | 607038 | *SLC7A8* | 604235 |
| *OTOF* | 603681 | *SMPX* | 300226 |
| *OTOG* | 604487 | *SNAI2* | 602150 |
| *OTOGL* | 614925 | *SOX10* | 602229 |
| *P2RX2* | 600844 | *STRC* | 606440 |
| *PAX3* | 606597 | *SYNE4* | 615535 |
| *PCARE* | 613425 | *TBC1D24* | 613577 |
| *PCDH15* | 605514 | *TBX1* | 602054 |
| *PDZD7* | 612971 | *TECTA* | 602574 |
| *PEX1* | 602136 | *TIMM8A* | 300356 |
| *PEX6* | 601498 | *TJP2* | 607709 |
| *PEX7* | 601757 | *TMC1* | 606706 |
| *PHYH* | 602026 | *TMEM132E* | 616178 |
| *PJVK* | 610219 | *TMIE* | 607237 |
| *PNPT1* | 610316 | *TMPRSS3* | 605511 |
| *PTPRQ* | 603317 | *TMTC2* | 615856 |
| *RDX* | 179410 | *TNC* | 187380 |
| *REST* | 600571 | *TPRN* | 613354 |
| *ROR1* | 602336 | *TRIOBP* | 609761 |
| *S1PR2* | 605111 | *TSPEAR* | 612920 |
| *SERPINB6* | 173321 | *TWNK* | 606075 |
| *SGO2* | 612425 | *USH1C* | 605242 |
| *SIX1* | 601205 | *USH1G* | 607696 |
| *SIX5* | 600963 | *USH2A* | 608400 |
| *SLC17A8* | 607557 | *WBP2* | 606962 |
| *SLC22A4* | 604190 | *WFS1* | 606201 |
| *SLC26A4* | 605646 | *WHRN* | 607928 |
| *SLC26A5* | 604943 |  |  |
