# Supplementary material for: A novel missense variant in MYO3A is associated with autosomal dominant high‐frequency hearing loss in a German family
Source: Mol Genet Genomic Med. 2020 Jun 10;8(8):e1343. doi: 10.1002/mgg3.1343 (PMC7434730; doi:10.1002/mgg3.1343)
Supplement: Supplementary file 3 — Table S2 [file MGG3-8-e1343-s003.docx]

**Table S2:** Summary of previously described recessive and dominant variants and the newly identified variant in *MYO3A*.

| Study | Variant | AD / AR | HL onset | HL severity | Ethnicity |
| --- | --- | --- | --- | --- | --- |
| Walsh et al. 2002 | c.732-2A>G **(kd)** | AR | second decade | moderate-to-profound, progressive | Israeli |
| Walsh et al. 2002 | c.1777-12G>A **(md)** | AR | second decade | moderate-to-profound, progressive | Israeli |
| Walsh et al. 2002 | c.3126T>G, p.(Tyr1042Ter) **(md)** | AR | second decade | moderate-to-profound, progressive | Israeli |
| Choi et al., 2013 | c.580C>A, p.(Pro194Thr) **(kd)** | AR | n.a. | n.a. | n.a. |
| Choi et al., 2013 | c.1588dupT, p.(Tyr530Leufs*) **(md)** | AR | n.a. | n.a. | n.a. |
| Miyagawa et al. 2013 | c.426T>G, p.(His142Gln) **(kd)** | AR | late-onset | n.a. | Japanese |
| Miyagawa et al. 2013 | c.848A>C, p.(Gln283Pro) **(kd)** | AR | early-onset | n.a. | Japanese |
| Miyagawa et al. 2013 | c.1324C>A, p.(His442Asn) **(md)** | AR | early-onset, late-onset | n.a. | Japanese |
| Miyagawa et al. 2013 | c.1819G>T, p.(Val607Phe) **(md)** | AR | early-onset | n.a. | Japanese |
| Wu et al. 2015 | c.4462A>G, p.(Lys1488Glu) | AR | n.a. | severe-to-profound | Chinese |
| Wu et al. 2015 | c.4681C>T, p.(Arg1561Ter) | AR | n.a. | severe-to-profound | Chinese |
| Sommen et al. 2016 | c.991C>T, p.(Arg331Ter) | AR | prelingual | moderate-to-profound | Western-European |
| Sommen et al. 2016 | c.1193C>A, p.(Ser398Ter) **(md)** | AR | prelingual | moderate-to-profound | Western-European |
| Qu et al. 2016 | c.1841C>T, p.(Ser614Phe) **(md)** | AR | congenital | profound | Kazakh |
| Grati et al. 2016 | c.1463G>A, p.(Gly488Glu) **(md)** | AD | early-onset | moderate-to-profound, progressive | African American |
| Dantas et al. 2018 | c.2090T>G, p.(Leu697Trp) **(md)** | AD | congenital, early-, late-onset | mild-to-severe, progressive | Brazilian |
| Present study | c.716T>C, p.(Leu239Pro) **(kd)** | AD | prelingual | moderate-to-profound, progressive | German |

Abbreviations: n.a., not available; AR, autosomal recessive; AD, autosomal dominant; md, motor-head domain; kd, kinase domain
